# Relationship Between Students’ Prior Academic Achievement and Homework Behavioral Engagement: The Mediating/Moderating Role of Learning Motivation

**DOI:** 10.3389/fpsyg.2019.01047

**Published:** 2019-05-08

**Authors:** Susana Rodríguez, José C. Núñez, Antonio Valle, Carlos Freire, María del Mar Ferradás, Carolina Rodríguez-Llorente

**Affiliations:** ^1^Department of Psychology, University of A Coruña, A Coruña, Spain; ^2^Department of Psychology, University of Oviedo, Oviedo, Spain

**Keywords:** homework, prior academic achievement, behavioral engagement, motivation, secondary education

## Abstract

The interest of assigning homework is frequently discussed due to its alleged low impact on student achievement. One of the current lines of research is to emphasize the quality of student homework engagement rather than the amount of time spent on homework. The aim of this study was to determine (a) the extent to which students’ prior achievement affects their homework engagement (i.e., time spent, time management, and amount of teacher-assigned homework done), and (b) how students’ intrinsic motivation toward homework may mediate or moderate the relationship between prior achievement and the homework engagement variables. A large sample of students from the first 4 years of Secondary Education (*N* = 1899) completed questionnaires. The results showed that intrinsic motivation partially mediates, but does not moderate, the effect of prior achievement on the three variables related to homework engagement (time spent, time management, and amount of teacher-assigned homework done). These results highlight the importance of considering both students’ current level of achievement and their motivation toward homework engagement when assigning homework.

## Introduction

Homework assignment is used regularly as an instructional strategy to optimize students’ learning and academic achievement (Cooper et al., 2006; Ramdass and Zimmerman, 2011). In general, there seems to be a positive relationship between homework and academic achievement (Trautwein et al., 2006; Núñez et al., 2015b; Fan et al., 2017), although this relationship will vary in magnitude and direction depending on variables such as students’ age, the amount of time spent, the management of that time, the motivational orientation or cognitive engagement, as well as the quality of parental involvement, or the quality of the teacher-assigned homework.

Current academic achievement, in turn, seems to be associated with student engagement in the future performance of homework. Moreover, based on the responses of a broad sample of students aged between 9 and 16 years old, Regueiro et al. (2015) found that prior achievement was significantly related both to students’ subsequent motivation to do homework (i.e., intrinsic motivation, interest, and perception of utility) and to their homework engagement (time spent on homework, homework time management, amount of homework done).

This relationship between prior achievement and homework engagement can be explained by different pathways, external (through parental or teacher involvement) and internal (different levels of knowledge, expectations of future achievement, perceived competence, motivation, etc.). From this point of view, students with good prior achievement may also meet the internal and external conditions that lead to favorable personal homework engagement, whereas if prior achievement is not good, the external and internal conditions will certainly not be as favorable for good homework engagement. Thus, for example, when family involvement becomes more controlling and there is lower motivational and emotional support (Núñez et al., 2015c, 2017; Regueiro et al., 2017a), teachers develop low expectations about the students’ engagement and future achievement (Kloomok and Cosden, 1994; Pitzer and Skinner, 2017; Zhu et al., 2018), and the students develop more negative expectations about their competence and future performance, and become discouraged and cease to engage progressively. These unfavorable affective-motivational conditions, in turn, are an added handicap to the already poor personal conditions (low academic achievement) when facing the next learning experiences (Ben-Naim et al., 2017). All of this often leads to a new academic failure, either partial (Klassen et al., 2008) or generalized to the entire academic area (Shifrer, 2016).

The present study analyzes the mediator or moderator role of intrinsic motivation regarding the effect of prior achievement on student homework engagement (time spent on homework, homework time management, and amount of homework done). Although there is abundant information available with regard to student engagement, the same cannot be said regarding the area of homework. The data from this study can contribute to better understanding the way in which past achievement can condition students’ future homework engagement.

### Prior Achievement and Motivation

Motivational variables determine student homework engagement; that is, students’ reasons for doing homework significantly influence their degree of engagement (e.g., time spent, optimization of that time, and amount of homework done) and their academic achievement (Pan et al., 2013).

However, the nature of the relationship between motivation and academic achievement is bi-directional, such that the latter is also a significant antecedent of relevant motivational factors in the academic field such as self-concept or self-efficacy (Marsh et al., 2005; Schöber et al., 2018). From this viewpoint, students’ learning failures, experienced not so much due to their skills as to their lack of motivation, lead them to developing beliefs of lack of competence, which, in turn, lead to low expectations of achievement and, as a consequence, low homework engagement and poor school performance. Therefore, the data derived from past research suggest including students’ prior achievement as an important variable to understand their homework engagement (Cool and Keith, 1991; Trautwein et al., 2002; Zimmerman and Kitsantas, 2005; Fast et al., 2010; Chen et al., 2013; Garon-Carrier et al., 2016).

A study carried out by Hong (2001) pointed out that high-performing students are more self-motivated to do homework than low-performing students. As a result, students who have already been successful in tasks like homework, compared to less successful students, feel more confident to perform tasks successfully in the future. Believing in their capabilities to achieve set goals influences students’ motivation and effort to learn and, therefore, their engagement (Schunk and Ertmer, 2000; Ormrod, 2003). In addition, academic achievement also maintains a positive relationship with other motivational variables, such as interest in the homework and the perception of its usefulness (Wigfield and Cambria, 2010).

### Motivation and Behavioral Engagement

The expectancy-value theory (Eccles et al., 1984; Wigfield and Eccles, 2000) is especially appropriate to explain the motivational aspects of behavior regarding homework (Trautwein and Köller, 2003). It indicates that students are more willing to engage in homework they perceive as emotionally rewarding and valuable, and where their effort is rewarded.

As shown in their work Ben-Eliyahu et al. (2018), we think about motivation as a pre-existing learner characteristic that produces engagement and self-regulated learning as part of engagement process. Schunk and Mullen (2012) describe this commitment as “the manifestation of students’ motivation.” Like various authors, Pekrun and Linnenbrink-Garcia (2012) suggest that commitment is a mediator between emotion and achievement, whereas Ainley (2012) argues that motivation leads to achievement through commitment. For other authors, motivation is a predictor of engagement (Lazowski and Hulleman, 2016) and, for Ben-Eliyahu et al. (2018), motivation triggers commitment. In previous studies, it was also found that different forms of motivation predict commitment (Patall et al., 2016; King and Datu, 2017).

Research suggests that students’ type of motivation for a task is significantly related to their engagement (Ryan and Deci, 2000). There is evidence indicating that many students do homework for extrinsic reasons, such as getting good grades, for their desire to please or to avoid punishment (Walker et al., 2004). However, this kind of motivation is associated with low levels of engagement, learning, and achievement (Vallerand et al., 1997). On another hand, students who perform homework driven by intrinsic reasons tend to show high levels of persistence, creativity, achievement, positive emotions, interest, and engagement (Flink et al., 1992; Bouffard et al., 2001; Coutts, 2004). Motivation is therefore considered a very influential variable in the process of doing homework and, specifically, in students’ homework behavioral engagement (Xu and Corno, 1998; Corno, 2000).

### Goal of This Study

Homework assignment without taking into account the diversity of the classroom is a habitual practice. This instructional strategy ends up being successful for some students, but is clearly inappropriate for others. Homework assignment should be adapted to the needs and potentials of the students. Otherwise, rather than helping them to develop, homework assignment progressively undermines their motivation and interest. In the present study, prior achievement and all that this entails (knowledge, perceived competence, expectations, etc.) were considered to constitute a potential determinant of student homework engagement (in terms of amount of time spent on homework, time management, and the amount of teacher-assigned homework done). In addition, we expect to answer the question of whether motivation mediates or moderates the relationship between prior achievement and homework engagement.

Therefore, we examined (a) the extent to which students’ prior achievement conditions their homework engagement, and (b) how students’ interest in doing homework (i.e., intrinsic motivation) may mediate and/or moderate that relationship. The initial hypotheses are as follows:

(1) Firstly, although the relation between time spent on homework and subsequent student achievement is clearly inconsistent (Cooper et al., 2006; Trautwein et al., 2006; Trautwein, 2007; Trautwein and Lüdtke, 2009; Dettmers et al., 2009; Fernández-Alonso et al., 2015; Núñez et al., 2015a,c), previous research argues that prior achievement significantly influences students’ academic engagement (e.g., Trautwein et al., 2002; Chen et al., 2013; Garon-Carrier et al., 2016). Under these precedents, it was hypothesized that the relationship between prior achievement and student homework behavioral engagement would be positive and statistically significant, suggesting that high-performing students would spend more time on homework, better optimize that time, and would do more teacher-assigned homework than low-performing students.
(2) Secondly, some data suggest that prior academic achievement positively influences students’ academic motivation (Valentine and Dubois, 2005; Schöber et al., 2018). In turn, students’ motivation is positively associated with the time spent on homework (Dettmers et al., 2009; Regueiro et al., 2015), the amount of homework done (Regueiro et al., 2017b), the management of homework time (Núñez et al., 2015a), and academic achievement (Valle et al., 2016). Therefore, we hypothesized that the relationship between prior achievement and student homework behavioral engagement would be partially mediated by students’ intrinsic motivation. In this way, intrinsic motivation would act as a mediator if the influence of prior achievement on student homework behavioral engagement were conditioned, at least partially, by the influence of students’ motivation. As well as the direct effect, the indirect effect of prior achievement on the variables of student behavioral engagement would also be positive (indicating that higher prior achievement is related to higher intrinsic motivation and greater student behavioral engagement).

Whereas mediation attempts to explain how and why certain effects occur, moderation provides information about when such effects will take place. In statistical terms, there is moderation when the interaction between the independent variable (in our case, prior achievement) and the third variable (intrinsic motivation) significantly affects the dependent variable (student behavioral engagement in homework). As there are no data from previous studies that have addressed this issue, we will not offer any hypothesis about the moderator role of intrinsic motivation. The question to explore here will be: is the effect of prior achievement on student homework behavioral engagement significantly different (e.g., in intensity or direction) as a function of students’ motivational level?

## Materials and Methods

### Participants

Participants were 1899 students (51.2% girls) of Compulsory Secondary Education (CSE) from 17 schools of four provinces in the north of Spain, of which 13 are public schools and 4 are subsidized. In terms of distribution by grade, 28.5% are enrolled in 1st grade of CSE (7th grade), 25.2% are in 2nd grade of CSE (8th grade), 22.2% are in 3rd grade of CSE (9th grade), and 24.1% are in 4th grade of CSE (10th grade). Participants’ age ranged between 12 and 16 years.

### Instruments

The variables time spent on homework, homework time management, amount of homework done, and homework intrinsic motivation were measured with several items of the *Homework Survey* (e.g., Núñez et al., 2015a,b,c; Valle et al., 2015a,b, 2018).

#### Time Spent on Homework

The students responded to two items (usually/during a typical week) with the following general formulation: “How much time do you usually spend each day on homework?” with the response options 1 = *less than 30 min*, 2 = *30 min to 1 h*, 3 = *1 h to an hour and a half*, 4 = *1 h and a half to 2 h*, 5 = *more than 2 h*. The reliability is acceptable (α = 0.78).

#### Amount of Homework Done

This information was obtained from students through their responses to two items related to the amount of teacher-assigned homework usually done. The two items were worded as follows: “Some students complete all their homework, and others only complete some of it. What about you? How much of your homework do you do…? (usually/during a typical week).” The students chose an answer from a 5-point Likert-type scale ranging from 1 (*I didn’t do any of my homework*) to 5 (*I did all my homework*). The reliability is acceptable (α = 0.82).

#### Homework Time Management

This was evaluated through the response to two items worded as follows: “Students often spend a lot of time doing homework, although most of the times, they don’t use that time properly, as they waste it (e.g., talking on the phone, being distracted by intrusive thoughts, procrastinating). And you, how do you manage the time you spend doing your homework (usually/during a typical week)?,” on which they were requested to rate their level of perceived quality of homework time management on a 5-point Likert-type scale ranging from 1 (*I don’t optimize it at all: “I am continually distracted by everything”*) to 5 (*I optimize it completely: I concentrate, and until I finish doing homework, I don’t think about anything else*). The reliability is acceptable (α = 0.77).

#### Intrinsic Motivation for Homework

Interest in learning by doing homework was assessed by students’ responses to eight items (*e.g., “I enjoy doing homework, because it allows me to learn more and more”; “Doing homework helps me understand what is being taught in class” and “Doing homework helps prepare me for the next day’s lesson/develop good self-discipline/learn how to plan my time or to be more responsible”*), which were rated on a 5-point scale ranging from 1 (*totally false*) to 5 (*completely true*). The reliability is acceptable (α = 0.86).

#### Prior Achievement

Prior achievement was evaluated according to the average academic grades obtained in the last year in Spanish, Math and foreign language (English). These grades were ranged from 1 to 5 (1 = insufficient, 2 = sufficient, 3 = good, 4 = notable, 5 = outstanding).

### Procedure

The procedure employed in this investigation followed the ethical standards of the Helsinki Declaration and was approved by the Research and Teaching Ethics Committee of the University of A Coruña. First of all, the prior written informed consent was obtained from the management team and the teaching staff of the participating schools. Subsequently, the written informed consent was obtained from the participants and their parents or legal guardians. Data collection was carried out during school hours. The instruments were administered by staff who collaborated in the research.

### Data Analysis

**FIGURE 1:**
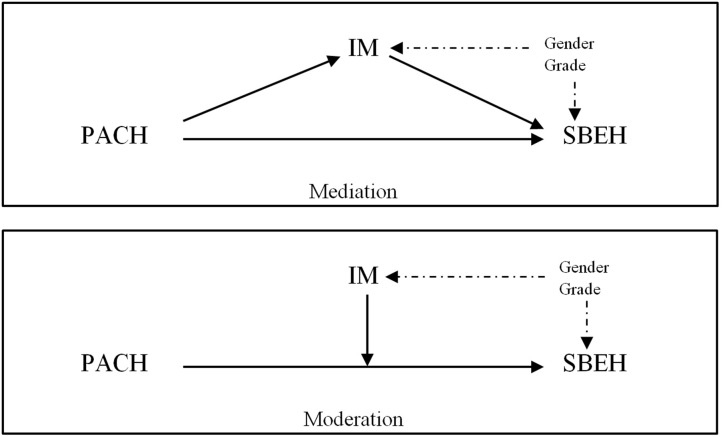
A simple mediation and moderation conceptual models of intrinsic motivation (IM) in the effect of prior achievement (PACH) on student behavioral engagement in homework (SBEH).

The data were analyzed with the SPSS 22 program. Twelve students were eliminated because they had a large amount of missing data or presented outlier values. No significant amount of missing data was found in any of the variables. The missing values were treated through the multiple imputation procedure. Prior to the study of the hypotheses, as preliminary analysis, we analyzed the correlation matrix and the distribution of the variables included in the study (prior achievement, intrinsic motivation, time spent on homework, time management, and amount of teacher-assigned homework done). With the help of the PROCESS (Hayes, 2013) module implemented in the SPSS, we analyzed whether intrinsic motivation mediated and/or moderated the effect of prior achievement on the three variables of student behavioral engagement considered. Figure 1 shows the mediation and moderation schema corresponding to hypotheses.

Gender and age (grade) were included in the design to statistically control for their potential effect. The effect sizes were calculated with Cohen’s (1988) *d*: *d* < 0.20 = minimum effect size; *d* > 0.20 < 0.50 = small effect size; *d* > 0.50 < 0.80 = medium effect size; *d* > 0.80 = large effect size.

## Results

### Descriptive Statistics

In Table 1 are summarized the descriptive statistics and Pearson correlations corresponding to the variables included in the study. The variables included in the study were significantly correlated, and the skewness and kurtosis data suggested an acceptable normal distribution. According to the relationship between the variables, we observed that: (i) females, compared to males, tended to spend more time on homework, reported better time optimization, and they did more assigned homework, had higher intrinsic motivation toward homework, as well as higher academic achievement; (ii) students’ motivation and interest and homework engagement decreased as they progressed through the school grades (7th to 10th grade); (iii) prior achievement had a significant and positive relationship with intrinsic motivation and student behavioral homework engagement; (iv) and homework time spent, homework time management, and amount of homework done were positively interrelated and positively related to intrinsic motivation.

**Table 1:** Descriptive statistics (mean, standard deviation, skewness, kurtosis) and Pearson correlation matrix.

	Gender	Grade	PACH	TSHW	TMHW	AHWD	IMHW
Gender	–						
Grade	0.037	–					
PACH	0.156^**^	-0.011	–				
TSHW	0.192^**^	-0.080^*^	0.128^**^	–			
TMHW	0.016	-0.158^**^	0.223^**^	0.168^**^	–		
AHWD	0.120^**^	-0.314^**^	0.352^**^	0.415^**^	0.384^**^	–	
IMHW	0.108^**^	-0.214^**^	0.189^**^	0.246^**^	0.368^**^	0.409^**^	–
M	1.510	4.420	2.790	3.140	3.220	4.079	3.440
SD	0.500	1.140	1.240	1.150	1.069	1.028	0.820
Skewness	-0.047	0.159	0.149	-0.088	-0.248	-1.121	-0.515
Kurtosis	-2.000	-1.397	-1.247	-0.798	-0.502	0.472	-0.043

*Gender (2 = females; 1 = males); Grade (3 = 7th; 4 = 8th; 5 = 9th; 6 = 10th); PACH, Prior Achievement; TSHW, Time Spent on Homework; TMHW, Time Management of Homework; AHWD, Amount of Homework Done; IMHW, Intrinsic Motivation toward Homework. PACH, TSHW, TMHW, AHWD, and IMHW (minimum = 1, maximum = 5). ^∗^p < 0.05. ^∗∗^p < 0.01.*

### Mediation Analysis

In Table 2 are summarized the results of the mediation analysis of the intrinsic motivation of the effect of prior achievement on student homework behavioral engagement (homework time spent, homework time management, and amount of homework performed).

**Table 2:** Summary of the mediation model.

	*Coeff.*	*SE*	*t*	*p*	*LLCI*	*ULCI*	*d*
**Homework intrinsic motivation**							
Constant	3.578	0.079	36.820	0.000	3.387	3.768	
Prior achievement	0.116	0.015	7.791	0.000	0.087	0.145	0.369
Gender	0.142	0.037	3.869	0.000	0.071	0.216	0.181
Grade	-0.152	0.016	-9.435	0.000	-0.184	-0.120	0.450
**Homework time spent**							
Constant	1.596	0.179	8.905	0.000	1.244	1.947	
Homework intrinsic motivation	0.290	0.033	8.893	0.000	0.226	0.354	0.423
Prior achievement	0.054	0.021	2.559	0.011	0.013	0.096	0.119
Gender	0.366	0.052	7.036	0.000	0.264	0.468	0.332
Grade	-0.033	0.023	-1.420	0.156	-0.078	0.012	0.066
**Homework time management**							
Constant	1.818	0.159	11.427	0.000	1.506	2.130	
Homework intrinsic motivation	0.418	0.029	14.452	0.000	0.362	0.475	0.714
Prior achievement	0.149	0.019	7.497	0.000	0.113	0.186	0.376
Gender	-0.094	0.046	-2.045	0.041	-0.185	-0.004	0.095
Grade	-0.068	0.021	-3.312	0.001	-0.108	-0.028	0.155
**Amount of homework done**							
Constant	2.958	0.136	21.681	0.000	2.690	3.225	
Homework intrinsic motivation	0.361	0.025	14.527	0.000	0.312	0.409	0.718
Prior achievement	0.237	0.016	14.712	0.000	0.206	0.269	0.729
Gender	0.123	0.040	3.106	0.002	0.045	0.201	0.145
Grade	-0.213	0.018	-12.114	0.000	-0.247	-0.178	0.588

*Gender (2 = women; 1 = men); Grade (3 = 7th; 4 = 8th; 5 = 9th; 6 = 10th); Prior achievement, homework time spent, homework time management, amount of homework done, and homework intrinsic motivation (scale: minimum = 1, maximum = 5); LLCI, lower confidence interval; ULCI, upper confidence interval.*

#### Mediation Model (Dependent Variable: Homework Time Spent)

The data obtained suggested that homework intrinsic motivation almost completely mediated the effect of prior achievement on homework time spent. Specifically, whereas the indirect effect of prior achievement on homework time spent was positive and statistically significant (*b* = 0.034, *p* < 0.001, *d* = 0.274), the direct effect was minimal (*b* = 0.054, *p* < 0.05), with a small effect size (*d* = 0.119). The overall effect was *b* = 0.088 (*p* < 0.001, *d* = 0.193). The mediational model explained 9% of the variability of the time spent on homework. The data also showed that gender was related to the prediction of time spent on homework (*b* = 0.366, *p* < 0.001), although the effect size was small (*d* = 0.332). Grade was not a predictor in this model.

#### Mediation Model (Dependent Variable: Homework Time Management)

Intrinsic motivation acted like a partial mediator of the effect of prior achievement on homework time management (indirect effect: *b* = 0.049, *p* < 0.001), although it had a small effect size (*d* = 0.323). Prior achievement also maintained a statistically significant but small direct effect on homework time management (*b* = 0.149, *p* = 0.001), (*d* = 0.186). The overall effect was almost intermediate (*b* = 0.198, *p* < 0.001, *d* = 0.486), explaining a total of 16.7% of the variability of homework time management. Gender and grade significantly predicted homework time management, although the effect size was minimal (no effect) (see Table 2).

#### Mediation Model (Dependent Variable: Amount of Homework Done)

The data provided by the mediational analysis indicated that intrinsic motivation was a partial mediator of the effect of prior achievement on amount of homework done (indirect effect: *b* = 0.042, *p* < 0.001), with a small effect size (*d* = 0.323). The direct effect was intermediate (*b* = 0.237, *p* < 0.001, *d* = 0.729), and the total effect was large (*b* = 0.279, *p* < 0.001, *d* = 0.841). The model explained 30.9% of the variability of the amount of homework done. Gender and grade were significant predictors, although whereas gender was hardly a predictor (*d* = 0.145), grade had an intermediate effect size (*d* = 0.588) (see Table 2).

### Moderation Analysis

Table 3 provides a summary of the moderation analysis of the intrinsic motivation of the effect of prior achievement on student homework behavioral engagement. The data derived from the analysis shows that intrinsic motivation does not have a moderating effect either in the relationship between prior achievement and time spent on homework (*b* = 0.002, *p* > 0.05, *d* = 0.003) or with homework time management (*b* = -0.004, *p* > 0.05, *d* = 0.007). This means that the effect of prior achievement on these two variables is of the same sign and intensity at any level of intrinsic motivation. However, a small moderator effect was observed in the relationship between prior achievement and amount homework done (*b* = -0.062, *p* < 0.01, *d* = 0.153). As can be observed in the last three rows of Table 3, depending on the level of intrinsic motivation, the effect size of prior achievement on amount of homework done was different in intensity (but not in direction). In general terms, the greater the intrinsic motivation, the lower the effect of prior achievement, and vice versa.

**Table 3:** Summary of the moderation of intrinsic motivation of the effect of prior achievement on student homework behavioral engagement (interaction effects).

Dependent variables	*Coeff.*	*SE*	*t*	*p*	*LLCI*	*ULCI*	*d*
Homework time spent	0.002	0.025	0.070	0.944	-0.047	0.051	0.003
Homework time management	-0.004	0.022	-0.160	0.873	-0.047	0.040	0.007
Amount of homework done	-0.062	0.019	-3.283	0.001	-0.100	-0.025	0.153
**Intrinsic motivation**							
2.628	0.290	0.023	12.731	0.000	0.245	0.335	0.620
3.449	0.239	0.016	14.857	0.000	0.207	0.271	0.737
4.269	0.188	0.022	8.535	0.000	0.145	0.231	0.405

*LLCI, lower confidence interval; ULCI, upper confidence interval.*

## Discussion

Doing homework is an instructional strategy frequently used by the vast majority of teachers, from all educational stages and all the countries belonging to the OECD. However, in the last report of this international organism, some concern was expressed about using this instructional strategy, as the data seem to indicate that countries using less homework are obtaining better achievement in PISA. They also indicated that the use of this strategy is negatively associated with children’s mental health. However, it is clear from the reviewed literature that the most rigorous studies suggest that such claims are not entirely true because other variables must be taken into account besides the time spent on homework, both extrinsic to the student (family involvement, teacher involvement) and those related to the students (level of prior knowledge, motivation, attitude, effort, self-regulation skills in the process of doing homework, etc.).

In this line, the present investigation sought to shed some light on this issue, focusing on the relative importance of the level of prior achievement in student homework engagement. Specifically, first, we studied the predictive capacity of prior achievement in student homework engagement in terms of the amount of time spent weekly, time management, and amount of teacher-assigned homework done. Secondly, we analyzed in greater depth how that relationship might be mediated, moderated, or both, by students’ intrinsic motivation (i.e., intention to engage in homework in order to learn and progress academically). The interest of the work was formulated in terms that if this relationship were significant, student’s current level of achievement should be taken into account by teachers when elaborating and assigning homework. And if motivation mediated or moderated the relationship, it should also be known and taken into account at this time. The main reason is that, if the hypotheses of the study were correct, the unadapted assignment of homework would be an inappropriate instructional strategy, partly responsible for students’ ambiguous relationship with achievement, and even for adverse consequences.

The results confirmed the first and second hypotheses, but not the third one entirely. These results will be discussed below in detail.

In the first hypothesis, we expected that the relationship between prior achievement and student behavioral engagement would be positive. The data partially confirmed this hypothesis. In particular, as expected, high-performing students, compared to low-performing ones, managed homework time better (although the effect size is small) and did more teacher-assigned homework (with an almost large effect size). On the contrary, the amount of time spent on homework was barely explained by students’ prior achievement (the size of the effect is practically non-existent). These results are in the line of those obtained in other studies, which also found that the amount of time spent on homework is of little interest (Trautwein, 2007; Dettmers et al., 2009; Regueiro et al., 2015).

The second hypothesis was also confirmed. In particular, it was found that the relationship between prior achievement and student homework behavioral engagement is partially mediated by students’ intrinsic motivation, indicating that higher prior achievement is related to higher intrinsic motivation and greater student behavioral engagement. As in other studies, the data from this research indicate that students’ motivation is positively associated, on the one hand, with academic achievement (Valle et al., 2016) and, on the other, with student homework engagement: the time spent on homework (Dettmers et al., 2009; Regueiro et al., 2015), homework time management (Núñez et al., 2015a), and the amount of teacher-assigned homework done (Regueiro et al., 2017b). This research found that the greater the prior achievement, the higher is students’ motivation and, finally, the greater their homework engagement. However, the amount of variance explained in each of the three variables of engagement is substantially different. Whereas only 9% of the time spent doing homework and 16.7% of time management are explained, 30.9% of the amount of teacher-assigned homework done is explained. But, while the data from this study refer to the importance of prior achievement and intrinsic motivation in the explanation of student homework engagement, they also raise some questions such as, for example, what personal variables are responsible for the amount of the remaining variance? what relevance do the family and school contexts have?

In terms of the moderation hypothesis, the results of the analysis of this study suggest that the effect of prior achievement on the time spent on homework and on time management does not change according to students’ motivational level. This means that the relationship described above has the same force and sign whether the student is little or very intrinsically motivated to work on homework. In the case of these two variables (time spent and time management), students’ motivation only facilitates an indirect pathway through which prior achievement would influence student homework engagement. However, some moderation was observed when the dependent variable was the amount of teacher-assigned homework done. In this case, and in general terms, when intrinsic motivation is high, the effect of prior achievement on the amount of homework done is smaller than when motivation is medium or low. These results can be interpreted in the sense that the higher the motivation, the lower is the capacity of prior achievement to determine student engagement in teacher-assigned homework. These findings offer a less deterministic vision: when students’ motivation is high, homework engagement is less determined by past conditions that we cannot influence. Therefore, high intrinsic motivation seems to be an important protective factor.

### Educational Implications

The results of this study have some implications for educational practice, which should be taken into account when designing and developing homework.

Firstly, we should assume that student homework engagement is determined by multiple factors that should be taken into account to ensure quality engagement. Students do not engage deeply in their homework just because it is their obligation (this may be the least powerful reason). As seen in this study, intrinsic motivation is an important determinant, mainly in terms of homework time management and the amount of teacher-assigned homework done, which in terms of the effect size, is close to large. As a result, and if only for this reason, it seems clear that it is not just is question of designing and assigning homework, but that homework and the contexts must be of quality, which invite the student to engage with them in order to learn. And it is not enough that the homework and the context are of quality, it is also necessary for students to perceive such quality so their deep engagement takes place (Rosário et al., 2018). Therefore in order to motivate students, an interesting practice when assigning homework might be to consider the relevance of each task with a view to students’ learning and personal autonomy.

Also, secondly, students’ prior achievement is shown as another important determinant of student homework engagement, mainly in terms of the amount of teacher-assigned homework done, and to a lesser extent, with regard to time management. However, as confirmed in the moderation analyses, in relation to the amount of homework done, this effect decreases when intrinsic motivation is high. Thus, insofar as we can highly motivate students to do homework with a deep focus, the determining effect of prior achievement will be lower and, therefore, low-performing students will be less vulnerable.

However, even in this case, it is relevant to take this into account when developing and assigning homework to the students. In general terms, from our data, poor achievement will lead to a decrease in intrinsic motivation (less interest in deep homework engagement), which will lead to a less effective behavioral engagement. In the end, this lower engagement could contribute to subsequent lower achievement, and so on. This loop would have obvious negative consequences. Therefore, it is necessary to significantly adapt the assignment of homework to this group of students, so that, taking into account these limiting initial conditions, the homework will involve real opportunities of personal engagement and success. This will facilitate student engagement – effective engagement – and, over time, the change of direction of that negative loop that makes them so vulnerable.

As previous research suggests, homework should be adapted to students’ potential and explicitly linked to academic success, but should also be perceived as useful by learners (Epstein and Van Voorhis, 2001, 2012; Trautwein et al., 2006; Trautwein and Lüdtke, 2009; Dettmers et al., 2010, 2011; Rosário et al., 2018). Teachers must face the challenge of linking homework characteristics to their students’ learning needs and interest. In this sense, it seems interesting that teachers explicitly state the competences and knowledge that is expected to be optimized with homework and that the instrumental, personal and/or professional use of the tasks that are sent home from the classroom are specifically agreed upon.

### Limitations

Although the results seem to be consistent, this research has some limitations that should not be ignored. Firstly, given that gender and grade were relevant in the explanation of student engagement, and although their effect was statistically controlled by including them as covariates, due to the characteristics of the statistical design, the data from this study do not provide information on how gender or grade might be moderating the effects found. Further studies could primarily examine this issue of undeniable relevance.

Secondly, it could be important to analyze the hypotheses of this study using data obtained with measurement instruments other than self-report measures, as this would allow us to determine the validity of the results of the scope of this study. Thirdly, would be of undoubted interest to study the objectives of this research in younger students, from Elementary Education, as the results of this research might not be generalizable to younger ages. Finally, although the procedure to study mediation/moderation is well established with data derived from cross-sectional designs, even with simple models of mediation/moderation, like those used in this investigation, the data obtained might have differed significantly if we had chosen a longitudinal data collection strategy (or repeated measures). For the design of future studies, this issue of particular relevance should be taken into account.

## Ethics Statement

This study was carried out in accordance with the recommendations of the Research and Teaching Ethics Committee of the University of A Coruña, with written informed consent from all subjects. All subjects gave written informed consent in accordance with the Declaration of Helsinki. The protocol was approved by the Research and Teaching Ethics Committee of the University of A Coruña.

## Author Contributions

SR, AV, CF, and MF collected the data and wrote the manuscript. JN analyzed the data and wrote the manuscript. CR-L collected the data and helped revision of the manuscript.

## Conflict of Interest Statement

The authors declare that the research was conducted in the absence of any commercial or financial relationships that could be construed as a potential conflict of interest.
